# Prosecution for Infanticide

**Published:** 1862-05

**Authors:** 


### PROSECUTION FOR INFANTICIDE.

Dr. H. Culbertson, of Zanesville, Ohio, in the "*Lancer & Observer*," after giving a condensed statement of the evidence in a recent trial for infanticide, offers the following valuable remarks.

We regret that our limits will not permit the full details of the case, with Dr. C.'s opinion, and its reasons, on the sanity of the respondent. The prisoner having been acquitted under cover of *insanity*—which from the report seems altogether gratuitous nonsense.

I. What is the true value of the hydrostatic test as a proof of breathing?

The principal objections to this test are: (*a*) That lungs may be artificially inflated; (*b*) that they may be inflated by the gas of decomposition; (*c*) that children may live for a time after birth without the intervention of apparent respiration; (*d*) that emphysema may cause lungs to float; (*e*) that hepatization and tuberculation of lungs are objections; (*f*) that pneumonia is an objection; (*g*) that atelectasis is an objection.

We will briefly consider these points:

(*a*) It is now established beyond a doubt that lungs may be partially inflated while *in situ*, and float on water on removal from the body. That lungs may be partially inflated when removed from the body, is too well known to need mentioning; but that they may be fully expanded artificially when *in situ*, is disproved by the researches of the most modern and reliable writers. It is even, as is well known by nine physicians out of every ten, extremely difficult to restore a still-born child artificially, with all the skill that the practitioner may exercise, and the no inconsiderable vitality of the recently born child. It seems, too, that artificial inflation may be confounded with partial natural respiration; and here lies one of the greatest objections to the hydrostatic test. Imperfect respiration may be mistaken for artificial inflation, because by the latter the lungs are never fully expanded. But this source of error is fortunately removed by remembering the fact that even the partial inflation of lungs *in situ* requires great skill in its accomplishment; and, furthermore, it is the height of improbability that in cases of infanticide restoration will be attempted, much less completed, by the suffering mother. On this point Taylor justly remarks: "One might be led to suppose that every woman tried for child-murder had made the praiseworthy attempt to restore a still-born child, although circumstances may show that she cut its throat, severed its head, or strangled it while the circulation was going on."

From these considerations we may then conclude that, if lungs are *fully expanded*, they are inflated from natural respiration, and have no fears of the objection of artificial inflation. But if lungs are partially inflated, we must look well to the condition of the child for other proof of live birth; and if there are no marks of violence, and no suspicious important circumstances attending the case, we may with the assistance of the other lights of the case—as the color and marbled state of the lungs, the development of the child and amplitude of the mother, the place in which the birth occurred, the consideration of the social relation of the mother after child shall have been born, and the absence of any congenital defect in the child—these will aid us in determining that artificial respiration was not performed, and the cause of the inflation. It, too, is improbable that any mother, good or bad, would think of restoring her child by artificial inflation, in her ignorance of such process, even if she desired most earnestly to preserve the life of the infant.

The next objection is—(*b*) That decomposition of lung-structure which, producing a gas, may cause these organs to float on water, is a well known fact, and it is equally true that this is an objection to the hydrostatic test. As a natural consequence, when putrefaction is advanced in the lungs we cannot depend upon this test. But if decay is merely upon the surface of the lung, especially if it is limited to a few points upon the superfices, and does not extend to the substance of these organs, and if, added to this, the substance of the lung is yet of natural consistence, not softened, we may safely conclude that putrefaction will not interfere with the value of the hydrostatic test in the case. We may still further be assured this view is correct, if, on firm pressure between cloths and thin boards, or between the fingers and cloths, the gas can be so far removed, without destroying the substance of the lung, as to cause the portion to sink in water. For it has been found, by actual experiment, that the gas of putrefaction can be so far removed from the lung by compression as to cause it to sink in water, while the air of respiration can not be forced from the lung so as to make it sink by any pressure short of that which would completly break down its structure, and often not then. The reason for this is obvious. The gas of decomposition is outside of the air-cells, in the cellular structure of the lung, while the air of respiration is within the air-vesicles, and can not be forced out except through the bronchioli, which result is not probable, as the latter, too, are compressed in the experiment; or by rupture of the air-cells, which must be a rare result in an undecomposed lung. While the gas of decomposition being carried around, the air-cells can be readily compressed out, as the cells of the cellular tissue more or less communicate with each other, and these with the external air.

Again, the objection of decomposition is considerably removed by the fact that decay takes place with great tardiness in the lungs—later than in any other organ, excepting the heart, uterus and bones. To illustrate this, I may cite one of my own experiments:

Nov. 12, 1861.—A small pig was placed in a manure pile, that decay might proceed as rapidly as possible. On the 15th, three days, it was half rotten. The lungs were not softened; the heart on surface showed some putrescent vessels. The lungs and heart together floated; on separating them, lungs sank (the animal never had breathed) and heart floated. On compressing the heart, it too sank.

This case not only illustrates how slowly decay progresses, but affords proof of the value of the hydrostatic test.

With these limitations and facts in view, we may consider the hydrostatic test safe, so far as limited decomposition is concerned; if decay be general through the lungs, the test is useless.

The next objection urged is—(*c.*) That life can long exist without respiration may well be doubted; that the new-born infant's heart may pulsate for a few moments, and there be no apparent respiration, is admitted as true. But what, we ask, have such cases to do with the true value of the hydrostatic test, since the pulsation of the heart must be shown by the mother or by another to have taken place? It follows, therefore, that if some party does not prove it, the evidence of life can not be shown in the case, and, consequently, life *being proven*, the hydrostatic test is not needed in such a case. The question then turns, not on the point, *Did* the child *breathe!* but, *Why did it not breathe!* To determine this, the state of the lungs and other organs are generally competent. This head will be again considered under ataletasis.

The next point is—(*d.*) It is asserted by certain writers that emphysema of lungs may be an objection to this test, but more modern authority believe that it can be, since those who consider it a source of error probably mistake the gas of putrefaction for the air of emphysema; and moreover it is difficult to imagine how we can have established the pathological condition of emphysema, without the lungs having been previously inflated with air. We may, then, with others, safely dismiss this objection.

Our next point is—(*e.*) That hepatization or tuberculation of lung is a well continued negative objection to this test may be doubted, because either of these conditions can be detected and distinguished from those evidences denoting breathing. Hepatization is rarely general; and if partial, some portion of the lung will float if breathing has been performed. If it is general, the child could not have breathed and the *hydrostatic test is relieved*, as the child could live but for a moment with such lungs. Tuberculation of lung is a structural disease that in its very nature will prevent the new-born infant living but for a few moments, and may be easily distinguished after death on examination. And if it be not in proof, no man in his right mind would presume such child could have supported independent life. We may narrate a case in point: We were recently called to a case of miscarriage at the seventh month, a natural labor. The surface of the child's feet and hands were discolored, and the cuticle upon the sides of its feet and on the back of several of its fingers separted on being handled. The abdomen was tumid and the scrotum distended with gas. The face and surface generally were dusky in color; but further than this we observed no marks of putrefaction. Notwithstanding these evidences of decay, this child was seen to breathe, but was not heard to cry. It breathed several times (jerking respirations) without aid, and eight or nine times by blowing in its face. When the cord ceased to pulsate it was tied and divided, and the child enveloded in cotton, excepting its face, after which it breathed several times by blowing in its face, when death took place.

*Post-Mortem.*—Twelve hours after death. Lungs unexpanded, of a dark liver color, containing points of hard white tissue. On placing heart and lungs on water together, and separate, and also a number of small portions, all sank.

Microscopic examination of these hardened points showed oil globules, colestrine, granuels, epithelial cells, the surface of which were granulated, and tubercle corouscles, which showed round nuclei with and without the aid of acetic acid.

Here then is a case in which a child was born and respiratory acts were observed, and yet the lungs showed no evidence of respiration, for the simple reason that they were so diseased as to obstruct the entrance of air into the air cells. It follows, therefore, that in strictness there was no respiration, and consequently the hydrostatic test can not settle the question of life. It is clear, too, that this infant had only negative, dependent life, and under the law would be ruled out as a case beyond its province. This subject will be indirectly referred to again under the head of atelectasis.

The next objection is—(*f.*) Pneumonia has also been urged as a negative objection to this test. It is well known, however, that this disease is extremely rare in new-born infants, and that when present in the very few exceptional cases, it can plainly be detected by its limitation and by the fact that portions of the lung are inflated. For my own part I have always believed that the use of an organ, as well as its disuse, may lead to corresponding diseases, and hence feel that the introduction of air into the lungs of the new-born infant may be a cause of disease, and consequently that the comparative state of rest of the intra-uterine lung is highly unfavorable to inflammatory disease of this structure before birth. We feel, therefore, that pneumonia is intimately associated with the presence of external air, and consequently doubt the accession of this disease without respiration having been established. We may, too, doubt its instantaneous production after birth, so speedily as be a source to of error in investigating cases of infanticide.

The next objection is—(*g*.) Atelectasis being simply a state of unexpanded lung, with no positive lung disease, we naturally conclude that the general system or some other organ of the infant must be at fault. It may be that the general powers of the infant's system are so weakened that the nervous influence is not sent down from the nervous centres with sufficient force to expand the chest, and therefore the lungs are not inflated; or it may result from a partial obstruction in the air passages. If this state result from partial obstruction, it may possibly be removed by the acts of inspiration and expiration, but if it be from lack of nervous power, or other than lung organic vital defect, the difficulty cannot be removed, and soon, a few hours at most, the child must die. It would seem, then, in these cases, that if on examining the air passages we find no obstructions, the possibility of such children assuming an independent state of existence is improbable. And if such can not assume independent life, we can not perceive how infanticide can be committed upon such infants, they being inevitably destined to perish for lack of respiration. It would be a misdemeanor to take the life of such cases, but not murder. But if the obstruction (as mucus) in the air passages were of such nature as to be removed by the effort of respiration, then infanticide could be inflicted on such infants, and it would be seen that the criminal, if guilty, should be held strictly accountable in such cases for the death of the particular child.

It is a fact, I believe, that children with atelectasis never recover, and can it be that our law will condemn the culprit for the murder of that which has no independent existence, and is destined never to possess such independence? But we do not know that a child laboring under this disease ever recovered, and until we can prove that they may recover, we feel that when the presence of this disease is shown we are bound to conclude the child could not have lived.

What are the obstructive causes which may prevent the expansion of the lungs and simulate atelectasis? 1st, strangulation by various means; 2d, hanging; 3d, suffocation; 4th, drowning.

It must be determined whether these (except hanging) are accidental or homicidal, by a careful examination of each and the attendant circumstances. Strangulation offers the greatest prospect of success, and suffocation next, for obvious reasons. The other means may be the homicidal measure.

The subject of atelectasis has lately been incidentally developed in the trial of Brock *vs.* Kellock, before Vice Chancellor Stewart (see January number of *London Lancet*, 1862), in the course of which trial Drs. Lee and Ramsbotham gave it as their opinion, that proof of respiration was, and Drs. Taylor and Tyler Smith that it was *not*, necessary to establish live birth; the last two gentlemen believing that circulation can, for a short time, proceed without respiration. Dr. Anstie, in commenting upon these opinions before one of the London medical societies, takes the still higher ground that *consciousness* in the infant commences with the process of respiration, and that until the infant has become *conscious* it can not be deemed to possess an independent existence; and that therefore consciousnes should be one of the great evidences of independent life in the new-born child. That consciousness follows respiratian is a fact which most physician's have observed. The child must breathe, then we hear the cry.

This doctrine we must believe is correct, but we doubt its practical application in these cases; for who, but the mother, in many such cases could say whether or not the child had cried? Yet if it be kept in view as a leading principle, it will certainly, so far as we can perceive, lead to truth.

The presence or absence of consciousness should be one of the tests in these cases, and the fact should be discovered if the *unconsciousness* could have been produced from other than lung disease, which may enable the enquirer to determine the probable viability of the child irrelative to respiration.

The doctrine of proof of respiration being required to substantiate live birth is, it seems to us, far more safe in law than the ordinary doctrine of live birth, and it merits the closest scrutiny of the medico-legal professions. The question must be closely considered in all its bearings. A large class of cases must prove beyond a reasonable doubt that so soon as respiration has been established, consciousness is a necessary sequence, before this doctrine can be adopted with safety. It, however, will not be correct to maintain because a new-born child is unconscious that it did not breathe, for other causes may render it unconscious. If such are not detectable on inspection, and if no proof can be afforded of respiration in the case, we are left without data, and should give the culprit the benefit of the uncertainty.

The conditiou of atelectasis may be readily detected on inspection, by the dark color and uninflamed state of the lungs, by their sinking in water, and by being readily inflated after removal from the chest, when they will readily float on water, thereby showing no organic changes in the lung structure.

Under this head of our subject we conclude.

1st. If during the performance of the hydrostatic test the lungs float high, especially if they are large, marbled and of a bright pink color, and if decomposition is not, or but to a limited extent is, present on the surface of the lung, we may consider this test reliable of breathing; and if of breathing, it is one thousand chances to one in favor of live berth.

2d. If decomposition is present in the substance of the lung, or over the entire surface, the hydrostetatic test is useless.

3d. If the lungs are partially inflated and there is no lung decay, the hydrostatic test may become useful in connection with the other phenomena of the case.

4th. That emphysema, pneumonia and lung tuberculosis cannot be serious objections to the validity of *this test.*

It seems, then, that the hydrostatic test stands at the head of all of our means for detecting breathing in the new-born child; and that it had not lost its ancient value, but has rather increased in importance, by the efforts of able persons, whose object has been to qualify and show the true value of the objections to the test. I need hardly say that we cannot generally depend on this test alone; but there are cases so palpable from this test alone, as to force the conviction of breathing. However, it is our duty as physicians to avail ourselves of every phenomenon in the case, considering this test as the most important, but remembering not to neglect any of the evidences.

It will be noticed that no use was made of the *static tests* in this case. This apparent neglect was not an oversight, but was omitted because we think but little dependence can be placed on either the positive or relative test. 1st. Because the weight of lungs before and after breathing differ largely in new-born infants; and 2d. Because the weight of the lungs to the body, before and after respiration is established, vary so intimately as to dictate the non-prediction of any facts from this source.

By reference to the testimony in this case, it will be seen that the physicians differed as to the value of the hydrostatic test. This may have been caused by some of these gentlemen understanding this test as simply referring to the fact of the lung floating on water; when in reality the test includes all those changes which are produced in the chest and lungs by respiration; as the enlargement of the chest, in depth and latterly, and of the lungs; the change in color and consistency of the latter, and the exudation of frothy mucus tinged with blood from incision of lung structure. The changes effected in the heart by respiration partially belong to this test; but the *static tests*, as they essentially differ in their nature from the *hydrostatic tests*, do not.

We now ask attention to the next division:

II. What is the proper duty of the physician in investigating these or other medico-legal cases? How far should he exert himself to obtain the truth relating to a particular case?

It must be evident that every sane man is interested in the maintenance of the public welfare, and therefore in furthering the ends of the law. No man can tell when he may need the protection of the law; when he least expects it he may be obliged to call for its magisterial aid. Society is but a unity for the development of good and happiness, and in order that it shall progress in the true sense of the term, each member must aid in the establishment of truth. The physician as a member of society has special duties to perform, as well as those of the citizen, and under no circumstances should he avoid these, provided the act will not conflict with his duties elsewhere, and he is competent, and he believes it will confer a benefit upon the community of which he is a member. No one will deny that, for a time, the physician may lay off his professional robes to act as the citizen, and again resume them; and I do not think any candid man will deny that a physician making a post-mortem examination, for and in behalf of the State, may for a time leave his profession, to inquire into extra-medical matter pertaining to a case. Why? Because he is both citizen and physician. Community expect of him, as his profession has given him additional mental power and scope, that he will use the same for their protection against social evils. And is it possible that we have among us a single physician who would not ferret out crime in any of its hydra forms, with all the ability he may possess? Is there one of us, feeling our true position and our duty to act, that would shrink from aiding the law, from a fear that his motives might be questioned? Where, if not from the observing physician, would many legal cases derive their support in open court? The physician may be cognizant of *extra*-professional facts as well as professional, and shall he not observe and report such? And if he may observe such facts without the pale of his calling, is it not his duty also to follow up the case and make suspicions reality, if they are "worthy the name of reality?" Nay, more, is there a physician, seeing suspicious circumstances in connection with a case, that would *dare* to make no effort to discover if they were not reality? How could such a man balance his account with society, if, turning on his heel, he left such a case to be developed as it might be? There is no half-way: the physician either owes a full duty to society, to eradicate crime to the utmost of his ability, or he owes no duty at all. He is either a member of the social sphere, or he is not.

In the performance of these duties, if he should be obliged to resort to cunning means to obtain his information, is he not the better qualified for his duty? But, to particularize, if called upon as a physician to examine a case of alleged infanticide, after having made a post-mortem, would he not properly endeavor to obtain further light in the case, by obtaining a physical examination of the supposed mother, that he might connect the one inquiry with the other; and if, after proceeding thus far, the thoughts of obtaining a confession should occur, or be suggested, should he not endeavor to obtain the same, not by threats or promises, but by persuasion? And when he saw by the manner and actions of the suspected party there was good prospect of obtaining such confession, he should continue his efforts, should he not be applauded as a faithful witness and an honest citizen? Such then are the circumstances of this case, and these remarks show all that we have to say under this head.

The spirit of these conclusions may be found in Dean and Taylor's *Medical Jurisprudence*. And a still more rigid course is pursued in some of the German courts (see *North American Review*, late No.) in the search of criminality.

Our next division is—

III. A consideration of the legal doctrine "live birth."

It will be perceived that none of the medical witnesses in this case testified that the hydrostatic test was an evidence of live birth in the legal sense.

Strange as it may appear, most legal authorities hold that the child must be entirely without the mother's parts to constitute birth, and this is qualified by some that the child must be capable of supporting an independent existence. One authority, we remember, holds that the child will be considered "born," although the cord may not have been divided after delivery. Physicians can not see the propriety of this definition. Doubtless it had its origin in a desire to be on the merciful side, ere punishment was inflicted, in a truly laudable attempt to give the prisoner the benefit of "*a doubt.*" But this principle of justice, however meritorious it may be, has, we fear, remotely favored the production of infanticide, for it is a notorious fact that but *very* few criminals are convicted of this offense.

It is, too, a dangerous position, because a child may be born *in part*, and murdered, and no *adequate* law can reach the case. Even were it shown by the admission of the mother, or other party present, that at such a stage of the labor the child was murdered, it would constitute no *capital* offense against the law.

But, it is maintained, the law must set a fixed line where labor ceases and external infant life begins; and this her edicts say is when the child shall have been expelled from the mother entirely. But this fixation is objectionable, knowing that we can determine the nature of the child's vitality, by showing the stage of the mother's labor; but hoping rather to demonstrate the stage of labor by showing the *vital character* of the child.

It is proved beyond controversion, that the child may, in some few cases, breathe before it is born in the legal sense, but these cases are exceptional. In such we have evidently an independent life commenced, yet not fully protected by the law. Take a case: a child's head is born, and a blow on that part feloniously killed it; the child is murdered, yet the law can not punish for murder, because it is not born in law.

The question soon turns practically to the point, Was *the* particular child criminally destroyed, or did it die naturally after such partial birth? How can we determine this question? We answer, if with the respiratory signs present we have those of asphyxia, and there are no marks of violence on the child, and the history of the case evinces a tedious labor, and its circumstances show no homicidal intention, and no fears need be entertained of conviction in such a case. It is not to such cases of infanticide that we refer, but to those not fully born, in which the child evidently breathed, and was plainly murdered, and yet the legal definition of live birth will effectually prevent adequate punishment. But there is yet another class of these cases that dictate the removal of this definition, viz: those negative examples where the child has been entirely born, but respiration so imperfectly established as not to be perceptible during infant life, or proven after death, and where the circulation proceeds at a feeble rate. According to *this* definition such infants live, when in reality they have no *independent life whatever*. It is true that such cases generally are in proof by other parties than the mother, and that they mostly die; but examples may occur unwitnessed, and the physician be called to prove that this child never lived, which he can not do, for the legal definition of live birth stares him in the face; for he is aware that although he can discover no respiratory signs of life, the child may yet have been "born alive" in the legal sense. These defects would seem to dictate a change in the law on this point, and what should this be? With great deference to what may be the opinions of others on this point, we suggest we have already alluded to it under the head of atelectasis, there holding that no infant can be deemed responsible, or to possess independent life, until *consciousness* has been established; and as we know this is not shown until respiration has begun, therefore the presence or absence of respiration in these cases should be the great test of live birth.

This certainly is safe doctrine for the mother in this class of cases, because we can conceive of no cause which will arrest birth and destroy the child, which would not show in or about the mother or child after the birth. According to this view, if the child does not breathe, it cannot require the protection of the law so far as infanticide is concerned; the question then turns to the crime of fœticide, which we are not considering. But to illustrate: If a child present with a wound upon its head, and the attendant circumstances show that in all probability the *mortal wound* could not have resulted from accident, and there are evidences of respiration having been established, and no congenital defects of the child which could produce *unconsciousness*, it is evident that consciousness following respiration was established, as this state is the known effect of respiration; and the criminal should be held responsible in such a case after it had been sifted in all its bearings.

In the fixation of this doctrine we have advanced in principle, yet it still remains to be shown in each case of alleged infanticide that the perils of labor may not have destroyed the child after consciousness was established, or that the want of consciousness may not have been produced from disease of the mother or child, or from accident. But these objectionable cases are rare, and if their rarity be insisted upon by the defense, and the *non-existence* of such be not proven by the prosecution, the exception does not prove the incorrectness of the principle, but only that it was not useful in the particular case, or that the prosecution did not properly develop the case.

The law claims that any motion (and crying includes motion) of the child is an evidence of life; but if the child should move its head ere the body was born, and it was observed, it would be no evidence of independent life, although obviously i might have been born alive and was murdered. The law, however, does not hold that crying is any more than an infantile motion, showing life, and does not point to its denoting the establishment of consciousness, which act ninety nine times in a hundred brings the conviction to us in the new-born infant of independent infantile life.

From these considerations we believe that consciousness should be the great test of the independent life of the child: prove this and the infant may be murdered and the criminal be responsible *for murder;* and if the principle is not established, we cannot see how murder of an independent being can be inflicted. From this it follows that the removal of the old doctrine of "live birth," and the substitution of that of "independent vitality," and the keeping in view that *without respiration* we cannot have *independent* infantile life, would further the ends of justice, and bo more safe, because more effectual, than the present law.

